# The mediating role of anxiety in the relationship between misophonia and quality of life: findings from the validated Turkish version of MisoQuest

**DOI:** 10.3389/fpsyg.2024.1361645

**Published:** 2024-04-16

**Authors:** Ezgi Ay, Mert Huviyetli, Eda Çakmak

**Affiliations:** ^1^Faculty of Health Sciences, Department of Audiology, Baskent University, Ankara, Türkiye; ^2^Department of Interdisciplinary Neuroscience, Ankara University Graduate School of Health Sciences, Ankara, Türkiye; ^3^Ear Institute, University College London, London, United Kingdom

**Keywords:** misophonia, anxiety, quality of life, MisoQuest, validity and reliability

## Abstract

**Introduction:**

Misophonia is a disorder characterized by decreased tolerance to certain sounds or their associated stimuli, and many measurement tools have been developed for its diagnosis and evaluation. The aims of the current study were to develop the Turkish version of MisoQuest, a fully validated misophonia questionnaire, to evaluate the relationships between misophonia, anxiety, and quality of life, and to examine the mediating role of anxiety in the relationship between misophonia and quality of life.

**Methods:**

The reliability of the Turkish version of MisoQuest was conducted using data from 548 participants (Mean age = 28.06 ± 9.36). Then, the relationships between misophonia, anxiety, and quality of life were evaluated in a separate sample of 117 participants (Mean age = 25.50 ± 6.31) using the State–Trait Anxiety Inventory (STAI) and the Short Form 36 (SF-36) questionnaire.

**Results:**

The results showed that the Turkish version of MisoQuest has good psychometric properties. Close-to-moderate positive correlations were found between misophonia and anxiety, and weak negative correlations were found between misophonia and quality of life. Anxiety mediated the relationships between misophonia and quality of life.

**Discussion:**

These results emphasize that misophonia may be an important problem affecting people’s quality of life and reveal the mediating role of anxiety on this effect.

## Introduction

Some individuals experience abnormal emotional, behavioral, and physiological reactions to specific everyday sounds (Ferrer-Torres and Giménez-Llort, 2022; Swedo et al., 2022). This is initially introduced as “Selective Sound Sensitivity Syndrome” (Bernstein et al., 2013), now commonly referred to as “misophonia” (Jastreboff and Jastreboff, 2001). Unlike sounds typically perceived as disturbing, individuals with misophonia suffer from decreased tolerance toward ordinary, innocuous, and mostly human-induced sounds such as chewing, sniffing, and breathing sounds (Schröder et al., 2013). However, stimuli that cause aversive reactions, also called “triggers,” are not limited to human-produced sounds. Studies have reported that all kinds of sounds, regardless of their source, as well as visual stimuli and repetitive movements, may trigger misophonic reactions (Dozier, 2015; Hansen et al., 2021). These reactions are irrespective of the physical characteristics of the stimuli (e.g., intensity or spectrum of sounds); instead, they are associated with various factors, including the individual’s psychological profile, previous experiences with the sound, and the context in which the sound is encountered (Jastreboff and Jastreboff, 2001).

The most common emotional responses to triggers experienced by individuals with misophonia include varying levels of anger, irritation, stress, anxiety, and disgust (Dozier, 2015; Jager et al., 2020). Besides these emotional reactions, sympathetic overactivity (fight-or-flight response) such as muscle tension, feeling of pressure in the chest, arms, and whole body, increase in heart rate and body temperature, physical pain and breathing difficulties have been reported (Edelstein et al., 2013; Dozier, 2015). Behavioral reactions involve coping strategies that individuals with misophonia use to reduce their exposure to trigger stimuli, such as escaping or avoiding situations where the trigger may be encountered, seeking to discontinue the triggering stimuli, and mimicking or reproducing the triggers (Edelstein et al., 2013). In extreme cases, verbal, or physical violence toward the source of the triggers has also been reported (Schröder et al., 2013; Tunç and Başbuğ, 2017). These maladaptive reactions can cause serious negative effects on sufferers’ social life, interpersonal relationships, performance of work or academic tasks, psychological status, and quality of life (Schröder et al., 2013; Jager et al., 2020).

There are different approaches in the literature that consider misophonia as a component of decreased sound tolerance, a symptom associated with various psychiatric disorders, or a new psychiatric disorder (Schröder et al., 2013; Jastreboff and Jastreboff, 2014). Different approaches also lead to differences in the criteria proposed for the diagnosis of misophonia. In the criteria published by Schröder et al. (2013), misophonic triggers are only sounds produced by other people, anger is the dominant reaction, and aversive reactions cannot be explained by other psychopathologies. Dozier et al. (2017) expanded these criteria and included all kinds of sounds and stimuli from different modalities as misophonic triggers and emphasized the immediate physical reflex response. Finally, Jager et al. (2020) expanded and updated the diagnostic criteria proposed by Schröder et al. (2013). Although there are various criteria proposed for the diagnosis of misophonia, there are no definitive criteria in international official diagnostic systems. Different questionnaires and measurement tools have been developed to assess the misophonia. The Amsterdam Misophonia Scale (A-MISO-S) developed by Schröder et al. (2013) and the Misophonia Questionnaire (MQ) developed by Wu et al. (2014) are commonly used tools in research and clinics. Recently developed scales for misophonia assessment include the MisoQuest (Siepsiak et al., 2020a), Duke Misophonia Questionnaire (DMQ) (Rosenthal et al., 2021), Selective Sound Sensitivity Syndrome Scale (S-Five) (Vitoratou et al., 2021), Berlin Misophonia Questionnaire-Revised (BMQ - R) (Remmert et al., 2022), and New York Misophonia Scale (NYMS) (Barahmand et al., 2023).

Differences in assessment and diagnostic criteria of misophonia are a barrier to generalizing findings, comparing results across studies, and estimating the prevalence of misophonia in the general population (Swedo et al., 2022). Considering misophonia as a significant social problem and conducting further research on its diagnosis and treatment seems essential for individuals’ social functioning and quality of life (Siepsiak and Dragan, 2019). MisoQuest developed by Siepsiak et al. (2020a) is a fully validated questionnaire developed to assess misophonia. MisoQuest was created based on the diagnostic criteria proposed by Schröder et al. (2013). Due to its small number of items and single-factor structure, it is considered a rapid and effective tool for screening misophonia. Therefore, the present study aims to evaluate the psychometric properties of MisoQuest in the Turkish population, to examine the relationships between misophonia and state–trait anxiety and health-related quality of life and to assess the mediating role of anxiety on the relationship between misophonia and quality of life. The findings will provide information on the generalizability of previous findings to different populations, contributing to a better understanding of the relationship between misophonia and anxiety and the impact of misophonic symptoms on individuals’ lives.

## Methods

### Participants

For the Turkish adaptation of MisoQuest, 548 participants aged 18–63 were included in the study. The mean age of the participants was 28.06 ± 9.36 (female = 27.26 ± 8.87, male = 31.19 ± 10.58). 20% of the participants were male (*n* = 111) and 80% were female (*n* = 437). The education level of 77% of the participants (*n* = 420) was college or above, and 23% (*n* = 128) was high school or below. The questionnaire was delivered to the participants online via social media applications. Participants with self-reported psychiatric diagnoses and hearing problems were excluded.

Following the validity and reliability study of the Turkish version of MisoQuest, data were collected from a different sample of 122 participants to evaluate the relationship between misophonia and state–trait anxiety and quality of life. The MisoQuest, State–Trait Anxiety Inventory (STAI) and 36-item Short-Form Health Survey (SF-36) scales were delivered online to the participants at this stage. Of the 122 people who participated in the study, three people were excluded because they stated that they were diagnosed with anxiety disorder and two people were diagnosed with panic disorder, and the study continued with 117 people. The mean age was 25.50 ± 6.31 (female = 24.87 ± 5.84, male = 29.18 ± 7.79). The demographic characteristics of the participants are given in Table 1.

**Table 1:** Demographic characteristics of participants.

	*n* = 548	*n* = 117
Age	*M* = 28.06, SD = 9.36 Range = 18–63	*M* = 25.50, SD = 6.31 Range = 18–46

	*n* (%)	*n* (%)
Gender		
Female	437 (80)	100 (86)
Male	111 (20)	17 (14)
Education		
High school and below	128 (23)	29 (25)
University and above	420 (77)	88 (75)
Marital status		
Married	186 (34)	31 (27)
Single	362 (66)	86 (73)

M, mean; SD, standard deviation.

### Measurement tools

#### MisoQuest

The MisoQuest is the first fully validated misophonia questionnaire with good psychometric values and excellent reliability (Cronbach’s alpha = 0.955). This scale, which includes a single-factor and 14 items, was developed by Siepsiak et al. (2020a) based on the misophonia diagnostic criteria published by Schröder et al., with some modifications. Unlike the criteria of Schröder et al., MisoQuest includes items related to all kinds of sounds, not only human-produced sounds. The scale only assesses aversive responses to sounds; sensitivities in other sensory modalities are not included. Each item has a 5-point Likert-type response category ranging from “1– Strongly disagree,” “2 – Disagree,” “3 – Undecided,” “4 – Agree” and “5 – Strongly agree.” There is no reverse item in the MisoQuest and the total score ranges from 14 to 70.

#### State–Trait Anxiety Inventory (STAI)

The STAI, developed by Spielberger et al. (1970), is one of the most commonly used tools to assess anxiety. It is a 40-item self-assessment questionnaire consisting of two subscales that provide separate measures of two components of anxiety: state and trait anxiety. The first twenty items measure situational or state anxiety (STAI-S), and the second twenty items measure underlying or trait anxiety (STAI-T). The State-Anxiety Scale evaluates how respondents’ feel about anxiety “right now, at this moment” through four scales: “1 - Not at all,” “2 – Somewhat,” “3 - Moderately so,” and “4 - Very much so.” The Trait-anxiety Scale assesses how people “generally feel” about anxiety with four scales: “1 Almost never,” “2 – sometimes,” “3 - Often,” and “4 - Almost always.” There are 10 reversed items on the state scale (items 1, 2, 5, 8, 10, 11, 15, 16, 19, and 20), and seven on the trait scale (items 21, 26, 27, 30, 33, 36, and 39). The range of scores is from 20 to 80, the higher the score indicating greater anxiety. In this study, the Turkish version of STAI adapted by Öner and Le Compte (1983) was used. The Cronbach alpha value reported by Öner and La Compte is between 0.83–0.87 for the Trait Anxiety Scale and between 0.94–0.96 for the State Anxiety Scale.

#### 36-item Short-Form Health Survey (SF-36)

The SF-36 is a standard measurement tool for assessing health-related quality of life and was developed by Ware and Sherbourne (1992). It is a Likert-type scale made up of 36 items, divided into eight dimensions: (i) Physical Functioning (PF), which assesses whether health conditions interfere with the ability to perform daily life activities; (ii) Physical Role Functioning (RP), which measures functional limitations due to health problems; (iii) Emotional Role Functioning (RE) which evaluates functional limitations by emotional problems; (iv) Social Functioning (SF), which impacts in quantity and quality of social activities induced by mental and physical problems; (v) Mental Health (MH), which measures aspects of depressive and anxiety; (vi) General Health (GH), which evaluates individual health status and its development tendency; (vii) Bodily Pain (BP), which measures degrees of pain to daily activities; and (viii) Vitality (VT), which is a subjective assessment of energy and tiredness. The scale has no total score; sub-dimension scores can range from 0 to 100, and higher scores mean better health status. The scores in these 8 domains can be reduced to two general components: The Physical Component Summary (PCS) score is calculated using the four physical health dimensions: PF, RP, BP and GH. The Mental Component Summary (MCS) score is calculated using the four mental health perceptions: VT, SF, RE and MH. In this study, the Turkish version of the SF-36 adapted by Koçyigit et al. (1999) was used. Cronbach’s alpha values reported by Koçyiğit et al. range between 0.73 and 0.76 for the subscales.

### Procedure

For the adaptation of MisoQuest to Turkish, written permission was first obtained from the developers of the questionnaire. MisoQuest, originally written in Polish, was translated from Polish to Turkish by a native Turkish translator who speaks Polish fluently. The Turkish translation was shared with the developers of the original version of MisoQuest, and a back translation into Polish was provided by a translator who speaks Turkish and Polish. An audiologist and a psychologist examined the Turkish translation of the questionnaire. Then, the questionnaire was applied to 5 individuals, and final adjustments were made in line with their feedback.

### Statistical analysis

For the item analysis of MisoQuest, corrected item-total correlation was used. Confirmatory factor analysis (CFA) was used to evaluate the construct validity of MisoQuest. CFA was performed using maximum likelihood estimation and robust versions of the fit indices because of the non-normal distribution of data. The goodness-of-fit of the model was examined with the ratio of chi-square value to degrees of freedom (*X*^2^/df), root mean square error of approximation (RMSEA), comparative fit index (CFI), and Tucker-Lewis Index (TLI). For these indices, the following cut-off values were used to indicate the goodness of model fit: *X*^2^/df ≤ 3, RMSEA≤0.06, CFI ≥ 0.95, TLI ≥ 0.95 (Hu and Bentler, 1999). Internal consistency was tested with Spearman’s rank correlation coefficient. Cronbach’s alpha coefficient and Spearman-Brown coefficient were used in reliability analyses. To evaluate normality of distribution Kolmogorov–Smirnov test was used. Pearson’s and Spearman’s rank correlations coefficients analysis were conducted to assess the relationship between misophonia and state anxiety, trait anxiety and quality of life. A value of *p* < 0.05 was considered statistically significant. The PROCESS macro for SPSS, a bootstrapping technique developed by Hayes (2017) was used to evaluate the mediating role of anxiety on the relationships between misophonia and quality of life. The number of bootstrap resamples was set at 5000. If the upper and lower limits of the 95% confidence interval did not include zero, the mediation effect was considered statistically significant. Statistical analyses were performed using R software (Version 4.3.3.), IBM SPSS 25.0, and IBM SPSS AMOS 25.0 programs (SPSS Statistics Version 25.0. IBM Corp., Armonk, NY).

## Results

### Item-total analysis

The corrected item-total correlation coefficients were between 0.49 and 0.70. These coefficients are shown in Table 2 for each item.

**Table 2:** Corrected item-total correlation and factor loadings.

Misoquest items (*n* = 548)	Corrected item-total correlation	F1	F2	F3
I1	0.60	0.66		
I2	0.58	0.63		
I3	0.63	0.66		
I4	0.62	0.56		
I5	0.55	0.59		
I7	0.70	0.56		
I6	0.64		0.47	
I8	0.60		0.54	
I9	0.55		0.64	
I12	0.64		0.63	
I14	0.50		0.46	
I10	0.66			0.46
I11	0.59			0.52
I13	0.49			0.64

F1, emotional reactions; F2, anger and avoidance; F3, functionality.

### Confirmatory factor analysis

**Figure 1:**
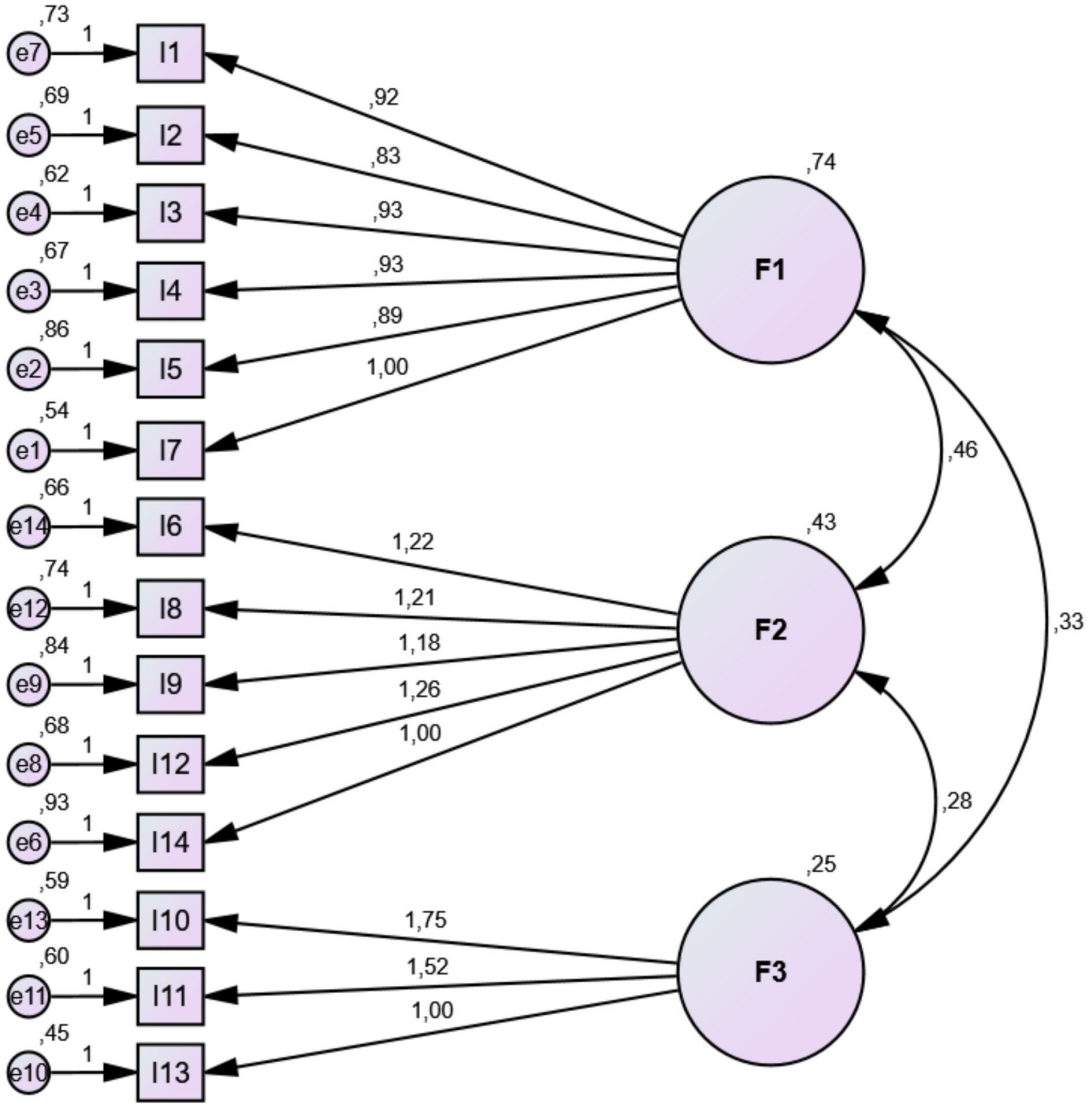
Confirmatory factor analysis model for MisoQuest.

First, the single-factor model of the Turkish version of the MisoQuest was evaluated. As a result of confirmatory factor analysis, the following goodness-of-fit indices were obtained: *X*^2^ = 338.02 (df = 77), *X*^2^/df = 4.39, RMSEA = 0.09, CFI = 0.89, and TLI = 0.87. These indices indicated a poor fit between the model and the data. A parallel analysis was conducted to determine the appropriate factor structure. This analysis suggested a 3-factor structure. For the 3-factor structure, the following goodness-of-fit indices were obtained: *X*^2^ = 200.45 (df = 74), *X*^2^/df = 2.71, CFI = 0.95; TLI = 0.94; RMSEA = 0.06. These indices indicated that the 3-factor model good fit between the model and the data. The goodness of fit values of the testing models are given in Table 3. The confirmatory factor analysis model for MisoQuest is given in Figure 1.

**Table 3:** Model fit indices for confirmatory factor analysis.

(*n* = 548)	$\chi^{2}\left(df\right)$	$\chi^{2}/df$	RMSEA	CFI	TLI
One-factor model	338.02 (77)	4.39	0.09	0.89	0.87
Three-factor model	200.45 (74)	2.71	0.06	0.95	0.94

$\chi^{2}/df\text{,}$ reduced Chi-square; RMSEA, root mean square error of approximation; CFI, comparative fit index; TLI, Tucker Lewis index.

### Factor loadings

Factor loadings for all items ranged from 0.46 to 0.66. The first factor (1.-5. and 7. items) was named “Emotional Reactions,” the second factor (6., 8., 9., 12., 14. items) was named “Anger and Avoidance,” and the third factor (10., 11., 13. items) was named “Functionality.” Factor loadings are shown in Table 2.

### Reliability analysis

According to the internal consistency analysis results, the correlations between the total score of MisoQuest and the sub-factors were significant and varies between 0.79 and 0.89. Correlation coefficients are shown in Table 4. The Cronbach’s alpha value for the entire questionnaire is 0.90. It was 0.85 for the “Emotional Reactions,” 0.79 for the “Anger and Avoidance,” and 0.72 for the “Functionality.” Spearman-Brown coefficient is found to be 0.92 for the whole MisoQuest and 0.86, 0.78 and 0.74 for the sub-factors, respectively.

**Table 4:** The correlation between the total score and sub-factors of the MisoQuest.

(*n* = 548)	Mean	SD	F1	F2	F3	MisoQuest total
**MisoQuest total**	36.33	10.51				
**F1:** Emotional reactions	15.91	5.16	–	0.639*	0.577*	0.891*
**F2:** Anger and avoidance	13.65	4.31		–	0.648*	0.882*
**F3:** Functionality	6.76	2.51			–	0.791*

*p < 0.001.

### Second order confirmatory factor analysis

After the first-order confirmatory model was confirmed, the second-order confirmatory factor analysis was conducted to identify whether all factors can be placed under a higher-order factor. This analysis reveals that three-factor measures a higher-order underlying construct, with very good reliability (0.84). The second-order latent variable was defined as “Misophonia.” Thus, it is possible to consider that the total score of MisoQuest measures a latent misophonia variable manifested through three sub-factors of MisoQuest.

### The relationship between misophonia and anxiety and quality of life

**Figure 2:**
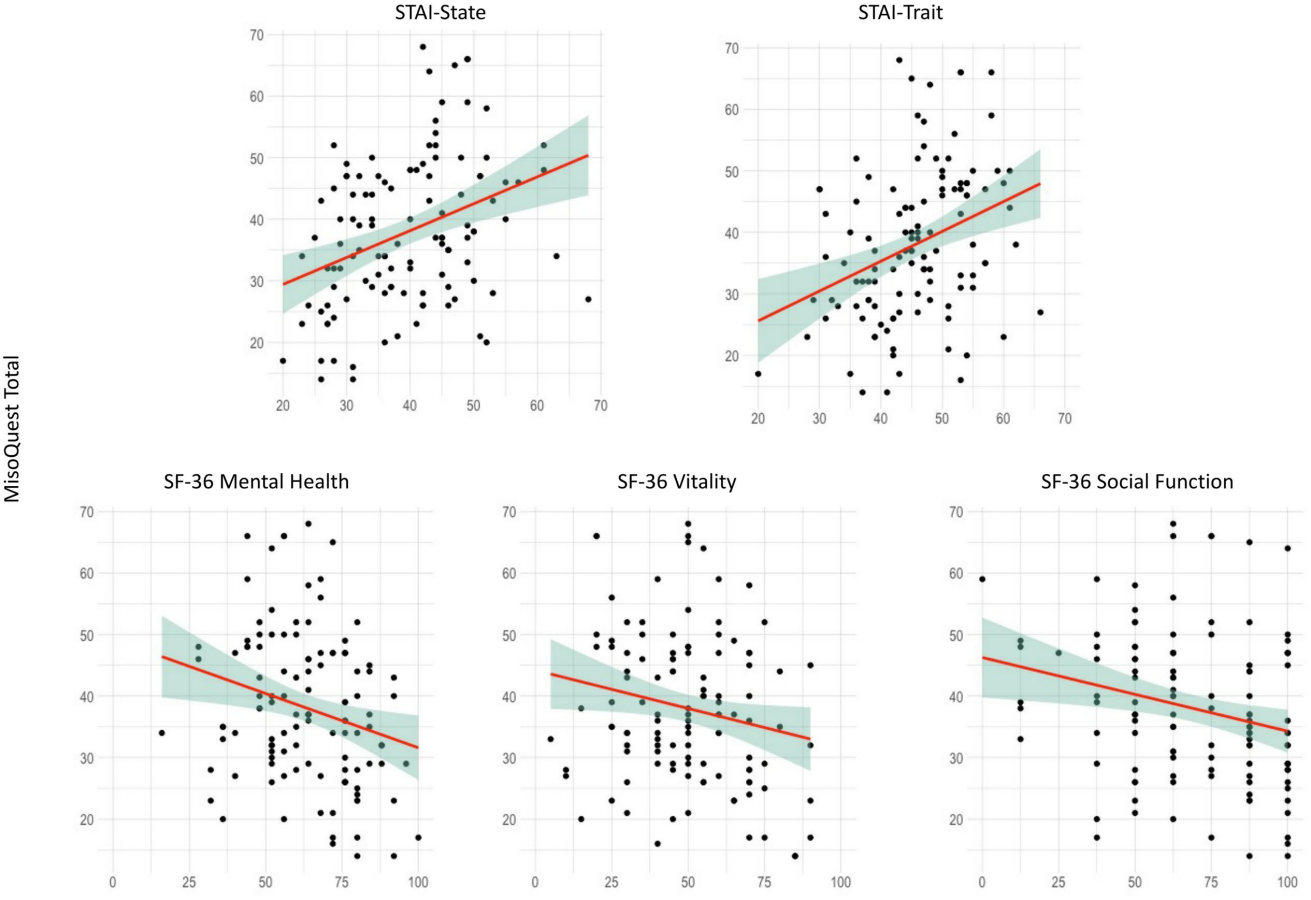
Scatter plots of correlations between MisoQuest with STAI and SF-36.

Statistically significant and close to moderate positive correlations were found between the MisoQuest total and sub-factors scores, and “STAI-State anxiety” scores (*r* = 0.349, *r* = 0.289, *r* = 0.384, and *r* = 0.286, respectively) as well as “STAI-Trait anxiety” scores (*r* = 0.334, *r* = 0.317, *r* = 0.335, and *r* = 0.247, respectively). Statistically significant weak negative correlations were found between the MisoQuest total, “Emotional Reactions” and “Anger and Avoidance” scores and “SF-36 Vitality (VT)” scores (*r* = −0.194, *r* = −0.182, *r* = −0.245, respectively), “SF-36 Social Functioning (SF)” scores (*r* = −0.242, *r* = −0.244, and *r* = −0.248, respectively), and “SF-36 Mental Health (MH)” scores (*r* = −0.241, *r* = −0.215, and *r* = −0.270, respectively). A significant weak correlation was found between the “Emotional Reactions” score and the “SF-36 Role Emotional (RE)” score (*r* = −0.183). The Pearson correlation coefficients between MisoQuest with STAI and SF-36 are given in Table 5. The correlation between the MisoQuest total score and other scales is shown as scatter plots in Figure 2.

**Table 5:** Pearson correlation coefficients between MisoQuest with STAI and SF-36.

(*n* = 117)	MisoQuest total	F1	F2	F3
STAI – State anxiety	0.349***	0.289**	0.384**	0.286**
STAI – Trait anxiety	0.334***	0.317**	0.335**	0.247**
SF-36 Physical functioning (PF)	−0.086	−0.151	0.006	−0.048
SF-36 Role physical (RP)	−0.101	−0.121	−0.074	−0.075
SF-36 Bodily pain (BP)	−0.043	−0.029	−0.091	−0.004
SF-36 General health (GH)	−0.154	−0.150	−0.154	−0.110
SF-36 Vitality (VT)	−0.194*	−0.182*	−0.245**	−0.050
SF-36 Social functioning (SF)	−0.242**	−0.244**	−0.248**	−0.123
SF-36 Role emotional (RE)	−0.176	−0.183*	−0.175	−0.119
SF-36 Mental health (MH)	−0.241**	−0.215*	−0.270**	−0.183

F1, emotional reactions; F2, anger and avoidance; F3, functionality.

*p < 0.05; **p < 0.01; ***p < 0.001.Bold font indicates significant correlations.

### Mediation model

**Figure 3:**
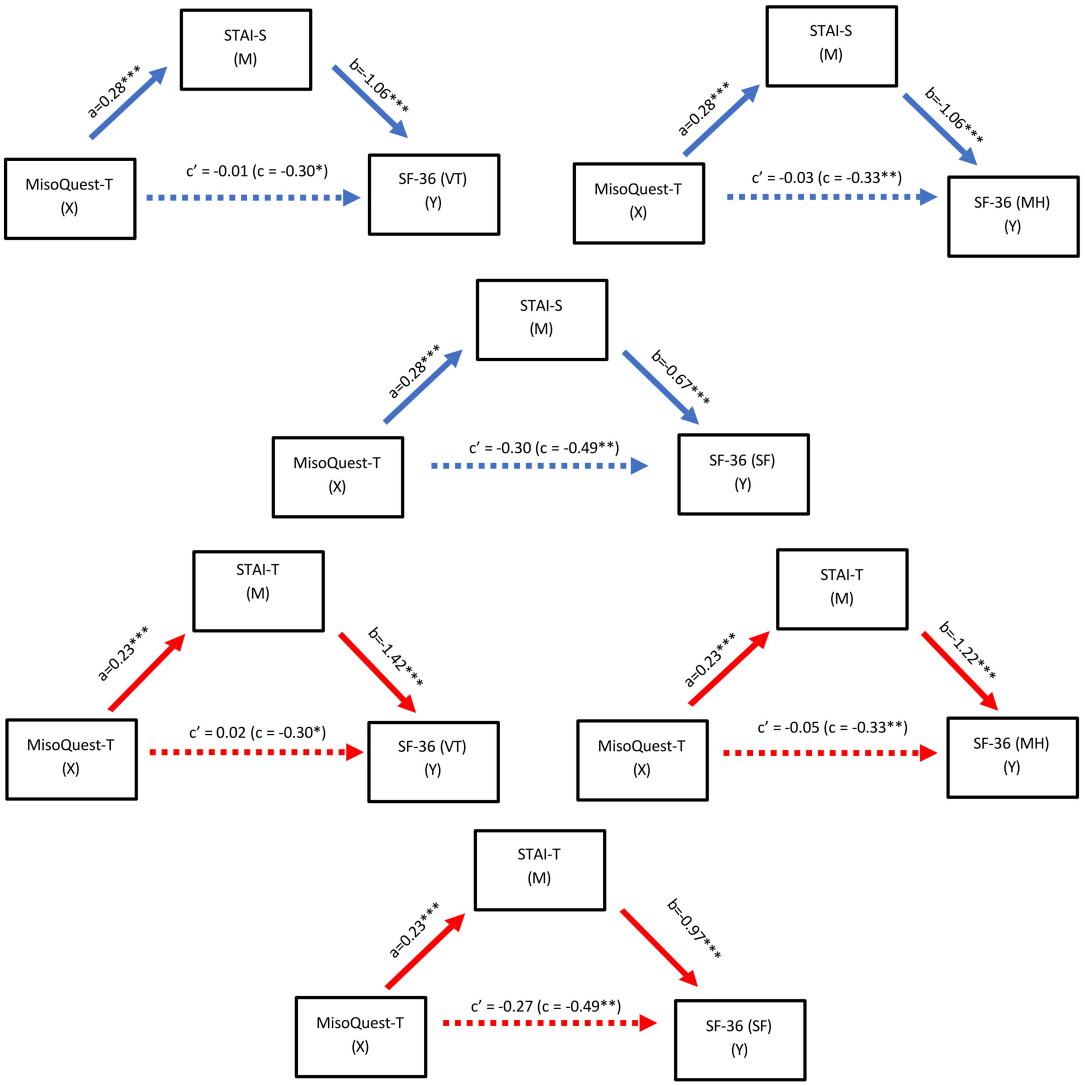
Path model of the mediating effect of state **(A)** and trait **(B)** anxiety on the relationship between misophonia and quality of life. Note. a,b,c, and c’ are unstandardised regression coefficients. a path (direct effect of MisoQuest-Total scores on STAI), b path (direct effect of STAI on SF-36), c (total effect of MisoQuest-Total on SF-36), c’ (direct effect MisoQuest-Total on SF-36). ^****^p < 0.0001, ^**^p < 0.01, ^*^p < 0.05.

The mediating role of state and trait anxiety on the relationship between misophonia and quality of life was examined separately. The MisoQuest Total score was determined as the independent variable and the three sub-factor scores of SF-36 [Vitality (VT), Social Functioning (SF) and Mental Health (MH)] that had a significant correlation with MisoQuest Total were determined as the dependent variable. For both state and trait anxiety, total effect of misophonia on SF-36 (VT), SF-36 (SF), SF-36 (MH) was statistically significant. The indirect effect of misophonia on SF-36 (VT), SF-36 (SF), SF-36 (MH) was also statistically significant. However, the direct effect of misophonia on SF-36 (VT), SF-36 (SF), SF-36 (MH) was not significant. These results suggest that state and trait anxiety fully mediated the relationship between misophonia and quality of life. The results of the mediation analysis are presented in Figure 3 and Table 6.

**Table 6:** Mediation of the relationship between misophonia and quality of life by state and trait anxiety.

*MisoQuest-Total - STAI-State - SF- 36(VT-SF-MH)*	*95% Confidence interval*						
		Effect	SE	t	*p*-value	Lower	Upper
	*Direct effect* MisoQuest-total ➔ SF-36 (VT)	−0.0053	0.1427	−0.0409	0.9675	−0.2640	0.2534
	*Indirect effect* MisoQuest -total ➔ STAI-S ➔SF-36 (VT)	−0.2971	0.0735			−0.4480	−0.1559
	*Total effect* MisoQuest -total ➔ SF-36 (VT)	−0.3025	0.1427	−2.1192	0.0362	−0.5852	−0.0198
	*Direct effect* MisoQuest -total ➔ SF-36 (SF)	−0.3026	0.1898	−1.5945	0.1136	−0.6786	0.0733
	*Indirect effect* MisoQuest -total ➔ STAI-S ➔SF-36 (SF)	−0.1882	0.0764			−0.3593	−0.0607
	*Total effect* MisoQuest -total ➔ SF-36 (SF)	−0.4908	0.1832	−2.6791	0.0085	−0.8537	−0.1279
	*Direct effect* MisoQuest -total ➔ SF-36 (MH)	−0.0309	0.1057	−0.2929	0.7701	−0.2403	0.1784
	*Indirect effect* MisoQuest -total ➔ STAI-S ➔SF-36 (MH)	−0.2973	0.0718			−0.4414	−0.1621
	*Total effect* MisoQuest -total ➔ SF-36 (MH)	−0.3282	0.1235	−2.6581	0.009	−0.5729	−0.0836
*MisoQuest-Total - STAI-Trait - SF- 36(VT-SF-MH)*	*Direct effect* MisoQuest -total ➔ SF-36 (VT)	0.0208	0.1222	0.1704	0.8650	−0.2213	0.2630
	*Indirect Effect* MisoQuest -total ➔ STAI-T ➔SF-36 (VT)	−0.3233	0.0869			−0.4960	−0.1565
	*Total effect* MisoQuest -total ➔ SF-36 (VT)	−0.3025	0.1222	−2.1192	0.0362	−0.5852	−0.0198
	*Direct effect* MisoQuest -total ➔ SF-36 (SF)	−0.2664	0.1849	−1.4409	0.1524	−0.6326	0.0998
	*Indirect effect* MisoQuest -total ➔ STAI-T ➔SF-36 (SF)	−0.2245	0.0913			−0.4257	−0.0697
	*Total effect* MisoQuest -total ➔ SF-36 (SF)	−0.4908	0.1832	−2.6791	0.0085	−0.8537	−0.1279
	*Direct effect* MisoQuest -total ➔ SF-36 (MH)	−0.0459	0.1052	−0.4366	0.6632	−0.2544	0.1625
	*Indirect effect* MisoQuest -total ➔ STAI-T ➔SF-36 (MH)	−0.2823	0.0758			−0.4359	−0.1369
	*Total effect* MisoQuest -total ➔ SF-36 (MH)	−0.3282	0.1235	−2.6581	0.009	−0.5729	−0.0836

## Discussion

A growing number of studies have attempted to understand symptoms of misophonia, its pathophysiology, prevalence, and relationship with different audiological, psychological, and psychiatric factors (Siepsiak and Dragan, 2019; Ferrer-Torres and Giménez-Llort, 2022). However, standard measurement tools used in clinics and research for the diagnosis and evaluation of misophonia are still lacking (Swedo et al., 2022). The purposes of the present study are to examine the Turkish psychometric properties of MisoQuest, to evaluate the relationships between misophonia, state and trait anxiety and health-related quality of life, and to assess the mediating role of anxiety in the effect of misophonia on quality of life.

Confirmatory Factor Analysis (CFA) revealed a three-factor structure for the Turkish version of MisoQuest. Since the factor loadings were greater than 0.3 and goodness-of-fit indices were good and quite acceptable in the CFA, the three-factor structure was considered appropriate. Reliability analyses indicate that the Turkish version of MisoQuest has high internal consistency. Cronbach’s alpha coefficient is very close to the value obtained in the original version of the questionnaire (*α* = 0.955) (Siepsiak et al., 2020a). Moreover, corrected item-total correlations indicate that each item contributes sufficiently to the total score. The results demonstrate that the Turkish version of MisoQuest is valid and reliable.

In the Turkish version of MisoQuest, items related to individuals’ internal emotional experiences, items related to anger response and avoidance of social environments, and items related to functionality in daily life are collected into three separate factors, unlike the original single-factor version of the questionnaire. A similar difference in factor structure between the original questionnaire and its Turkish version was also reported in the MQ (Wu et al., 2014; Sakarya and Çakmak, 2022). It is possible that the experience and expression of misophonia may vary as a result of social and cultural norms in different societies. Zhou et al. (2017), reported weaker correlations between misophonia symptoms and impairment in functioning in Chinese students than in American students. Turkey is considered a collectivist country in terms of cultural values such as commitment to the group and family (Kağitçibasi, 1996) and is similar to China in this context (Suh et al., 1998). Obtaining a different factor structure from the original questionnaire in this study may be due to these characteristics of Turkish culture. Studies that evaluate different cultures with the same standard measurement tools will increase our knowledge of the cross-cultural characteristics of misophonia.

Due to differences in assessment methods, measurement tools and diagnostic criteria, the frequency of misophonia reported in studies varies within a wide range between 3 and 55% (Jastreboff and Jastreboff, 2014; Wu et al., 2014; Zhou et al., 2017; Naylor et al., 2021). In a study conducted with depression patients using MisoQuest, it was reported that 8.5% of the patients met the misophonia criteria both in face-to-face interviews and in MisoQuest (when the cut-off value was 61 and above) (Siepsiak et al., 2020b). In another study conducted with 253 individuals using the same cut-off value, 69% of participants had self-reported misophonia, while 45% of those reporting self-reported misophonia met the MisoQuest cut-off criterion (Enzler et al., 2021). In our study, misophonia was detected in 2% of the total sample (N = 665) according to this cut-off value. Although this percentage is lower than those reported in other studies, it is close to Jastreboff and Jastreboff’s (2014) estimates of 3.2%. It is thought that the higher frequencies of misophonia reported for different measurement tools may be related to the fact that these tools include more general sound sensitivities and lower severity misophonia symptoms in measurement (Siepsiak et al., 2020b). Individuals with misophonia find similar stimuli to be aversive to those without misophonia, but they experience extreme levels of aversion (Edelstein et al., 2013). Additionally, when only severe symptoms are taken into account, the misophonia frequency reported in the studies decreases considerably (Naylor et al., 2021). MisoQuest appears to detect more severe and quality of life impairing levels of misophonia, consistent with the purpose for which it was developed (Siepsiak et al., 2020b). Evaluations using different misophonia measurement tools on the same population may provide comparable findings regarding these tools.

Although misophonia is considered a separate disorder with unique clinical features and neurophysiological mechanisms, many studies are reporting a relationship between misophonia and different psychiatric symptoms and psychopathologies (Schröder et al., 2013; Erfanian et al., 2019; Jager et al., 2020). Two studies conducted with similar methodology on samples from different cultures found significant positive correlations between misophonia symptoms assessed by MQ and anxiety, depression, and OCD symptoms (Wu et al., 2014; Zhou et al., 2017). In patients with depression, anxiety was found to be more highly correlated with the severity of misophonia measured with MisoQuest than other variables (Siepsiak et al., 2020b). Additionally, studies have shown that anxiety has a mediating role on anger outbursts associated with misophonia (Wu et al., 2014; Zhou et al., 2017). In our study, a significant, close to moderate positive correlation was obtained between MisoQuest scores and STAI-State and STAI-Trait anxiety scores. Our findings also show that anxiety has a mediating role in the relationship between misophonia and quality of life. This finding indicates that as the severity of misophonia increases, individuals’ anxiety levels would increase as well, and the deterioration in their quality of life may be a function of this increase in anxiety levels. Many studies report that exposure to misophonic triggers causes anxiety symptoms (Edelstein et al., 2013). However, some studies report that exposure to these triggers elicits anticipatory anxiety associated with thinking about future misophonic situations rather than eliciting an immediate anxiety response (Jager et al., 2020). Based on the relationship between misophonia and anxiety, there are studies that suggest anxiety treatment approaches in the treatment of misophonia (Bernstein et al., 2013). Randomized controlled clinical studies are needed to better understand the causal relationship between misophonia and anxiety. These studies may also provide a better evaluation of the effectiveness of anxiety-based intervention in misophonia.

Negative reactions caused by misophonia can cause deterioration in both interpersonal relationships and work or academic task performance, and can remarkably affect the person’s well-being, daily functionality, and quality of life (Edelstein et al., 2013; Wu et al., 2014; Zhou et al., 2017; Ferrer-Torres and Giménez-Llort, 2022). The impact of misophonia on an individual’s quality of life can range from a mild effect to severe impairment. The greater the severity of misophonia, the greater the impact on individuals’ quality of life (Jager et al., 2020). In our study, individuals’ quality of life was evaluated with the SF-36 scale, and significant negative correlations were found between the misophonia total scores and the Vitality (VT), Social Functioning (SF) and Mental Health (MH) factors of the SF-36. The Role Emotional (RE) factor of SF-36 was found to be correlated with the “Emotional Reactions” factor of MisoQuest. These findings support the previous findings that have shown the negative impact of misophonia on an individual’s quality of life. Moreover, no correlation was found between S-36’s physical health-related factors, Physical Functioning (PF), Role Physical (RP), Bodily Pain (BP), General Health (GH), and misophonia levels. Although some people with misophonia report that they also experience unpleasant sounds as painful, the comorbidity of misophonia with any somatic problems has not been reported (Rosenthal et al., 2022). Our findings support the previously reported view that misophonia is perceived as a mental problem rather than a physical problem (Kılıç et al., 2021). However, this finding may be related to the fact that the majority of our sample consisted of normal individuals without a diagnosis of clinically significant misophonia.

### Limitations

There are several limitations in this study. First, using the online self-report method in data collection may cause recruitment bias. Second, participants’ psychological and audiological diagnoses were based on self-report. Evaluations made by clinical psychologists, psychiatrists, and audiologists experienced in misophonia will be important in subsequent studies, especially in detecting comorbid disorders. Third, the age, educational status, and gender distribution of the individuals in the sample was not balanced. This may affect the generalizability of the results. Fourth, the target sample of this study was individuals from the normal population, but case–control studies including individuals clinically diagnosed with misophonia will contribute to a better understanding of the differences between individuals with misophonia and the normal population.

## Conclusion

An increasing number of measurement tools have been developed for misophonia in recent years, and adapting these tools to different cultures is essential in providing a standard measurement for misophonia and revealing intercultural differences. In this study, the validity and reliability of the MisoQuest in Turkish was evaluated, and it was shown that the Turkish version of the questionnaire has good psychometric properties. Developing new measurement tools with good psychometric properties for the diagnosis of misophonia and ensuring cross-cultural adaptation of these tools will make significant contributions to the creation of a standardized diagnosis and treatment protocol for misophonia. We believe that the Turkish version of MisoQuest will be a useful measurement tool that can be used both as a screening tool in clinical practice and in misophonia research. To our knowledge, our study is the first to evaluate the relationship between misophonia assessed with MisoQuest and anxiety symptoms and quality of life in the normal population. Our study found a significant relationship between the misophonia level assessed with MisoQuest and people’s state and trait anxiety levels and their quality of life.

## Data availability statement

The original contributions presented in the study are publicly available. This data and Turkish version of MisoQuest can be found here: https://doi.org/10.6084/m9.figshare.24903288.v2.

## Ethics statement

The studies involving humans were approved by the Baskent University Clinical Research Ethics Committee (Project number: KA21/72). The studies were conducted in accordance with the local legislation and institutional requirements. The participants provided their written informed consent to participate in this study.

## Author contributions

EA: Writing – review & editing, Writing – original draft, Validation, Project administration, Methodology, Investigation, Formal analysis, Conceptualization. MH: Visualization, Writing – review & editing, Writing – original draft, Validation, Methodology, Investigation, Formal analysis, Conceptualization. EÇ: Visualization, Data curation, Writing – review & editing, Writing – original draft, Formal analysis.
